# Do maternal cells trigger or perpetuate autoimmune diseases in children?

**DOI:** 10.1186/1546-0096-5-9

**Published:** 2007-05-16

**Authors:** Anne M Stevens

**Affiliations:** 1 Department of Pediatrics, University of Washington, Children's Hospital and Regional Medical Center, 307 Westlake Ave N, Suite 300, Seattle, WA 98109, USA

## Abstract

The placental barrier is not the impenetrable wall that it was once presumed to be. During pregnancy, fetal cells pass into the mother, where they persist for decades after the pregnancy, leading to ***fetal microchimerism *(FMc**). Maternal cells also pass into the fetus, where they can persist long after birth of the child into adulthood, leading to ***maternal microchimerism*(MMc**). FMc and MMc represent foreign cells, and thus have been implicated in the pathogenesis of autoimmune diseases that resemble graft-versus-host disease after stem cell transplantation. FMc, hypothesized to contribute to the high predisposition of autoimmune diseases in women, has been reviewed recently. In patients who have never been pregnant, (children, males, and nulliparous females), MMc may represent the foreign cells that initiate or perpetuate chronic inflammatory disease.

## 

### Is persistent maternal microchimerism (MMc) normal in infancy?

In human pregnancy, cell traffic during pregnancy is bi-directional, with maternal cells passing into the fetal circulation and fetal into the maternal. Maternal cells can engraft in infants with severe combined immunodeficiency (SCID), unbothered by a defensive host immune system [1-7]. In a large cohort of infants with SCID, MMc was detected in 40%, and graft-versus-host disease (GVHD) developed in 76% [7]. Both maternal T and B lymphocytes have been described engrafting into immunodeficient infants [1,3-7], but unlike stem cell transplantation, when donor cells functionally replace the host immune system, maternal cells physically replace but do not function for the child's immune system. The functional capacity of chimeric maternal cells is not well-defined. *In vitro*, chimeric maternal cells respond poorly to specific mitogens such as antigen or allogenic stimulator cells [1,5,6]. Chimeric maternal T cells do respond to non-specific mitogens such as IL-2, anti-CD3 antibodies or PHA, and maternal cell lines have been grown from the blood of immunodeficiency patients. These maternal cell lines were able to proliferate normally and express maternal HLA molecules [4-7]. Although T lymphocyte activation markers have been detected on chimeric maternal lymphocytes and MMc has been associated with GVHD, 40% of patients with detectable MMc never developed GVHD, suggesting that regulatory mechanisms control the engrafted maternal lymphocytes. One reason for the limited ability of maternal T lymphocytes to respond to specific antigens may be the limited T cell receptor repertoire in engrafted maternal cells [6]. Thus, a limited number of maternal T lymphocyte clones may be transported into the fetus and expand in response to non-specific stimuli. On the other hand, a random selection of maternal cells may travel to the fetus and specific clones expand by an antigenic stimulus yet to be identified, leading to clonal over-representation in the total population. The rate of persistent MMc in SCID patients is not known, because mortality is high, and most patients now receive stem cell transplants.

Whereas in SCID infants maternal cells make up the majority of lymphocytes, MMc is found at lower levels in immunocompetent infants. Maternal cells have been estimated at a rate of 0.02–5% in cord blood [8-10]. Early studies detected MMc in cord blood by labeling maternal blood cells with a fluorescent dye or Chromium-51 and injecting them back into the mother hours before delivery [11,12]. In non-invasive studies using fluorescence *in situ *hybridization (FISH) with probes to the X- and Y-chromosomes, maternal cells with two X-chromosomes were found in 20% of cord blood samples from male infants [8]. MMc was found in both the CD8+ and CD34+ subsets, suggesting the transfer of maternal stem cells. In studies using more sensitive polymerase chain reaction (PCR)-based assays, maternal DNA was found in 24–100% of cord blood samples [10,13-15]. Maternal DNA has been reported in the fetal circulation as early as 13 weeks gestation in blood samples taken prior to elective terminations [9,10]. By 20–33 weeks gestation, 53% of fetal blood samples harbored MMc [16]. Lo, et. al. developed quantitative techniques to study bi-directional traffic, and found maternal-to-fetal cell transfer was common, though less frequent than fetus to mother, and at lower levels [10].

### Is persistent maternal microchimerism (MMc) normal after infancy?

Maternal cells derived during gestation are not necessarily eliminated by the child's immune system early in life, but can persist into adult life. The original study suggesting that MMc can persist long after birth used PCR for non-inherited non-shared maternal HLA alleles[17]. In men, FISH for X- and Y-chromosomes was also used to detect female cells (presumed to be maternal). By these two methods MMc was detected in 55% of subjects, as young as nine years old and as old as 49 years. It has since been confirmed by others that a low level of maternal cells can create a state of MMc in the child persisting for decades [18,19]. The levels of MMc in these studies were only roughly estimated until Lo and Lambert, et. al., developed a panel of real-time quantitative PCR (Q-PCR) assays specific for highly polymorphic HLA alleles that could be used to accurately measure a low level of maternal DNA by targeting maternal HLA alleles not shared by the child [10,20]. Eight different assays were originally shown to be specific by testing on a panel of HLA-specific cell lines, and to have the sensitivity to detect one genome equivalent (gEq) of chimeric DNA in 100,000 gEq of host genomic DNA. By assaying genomic DNA isolated from peripheral blood mononuclear cells, evidence for MMc was found in 22% of healthy females aged 13–62 years. The levels of MMc ranged from 0 to 55 gEq/million host gEq. Thus, MMc appears to be common in healthy children and adults.

### What are the phenotypes of chimeric maternal cells?

The phenotypes of maternal cells in the blood, however, were not known until Loubière, et. al. assayed MMc in genomic DNA isolated from blood cell subsets sorted by flow cytometry. [21] By the same Q-PCR assays, maternal cells were found with slightly increased frequency in subsets, suggesting that they may be concentrated in one cell line or another. The levels of MMc were higher than levels in total peripheral blood cells, rising to as high as 360 per million in T lymphocytes, B lymphocytes, monocytes and natural killer cells. Most subjects with MMc in at least one cell subset did not have detectable MMc in unfractionated peripheral blood mononuclear cells. That MMc was present in every hematopoietic cell subset tested suggests that a maternal stem cell may engraft into the fetus, able to renew multiple cell lineages throughout the life of the child. What controls the level of MMc is not known. Pregnancy may affect the level of MMc, and may partially explain the lack of MMc in the younger women studied, who may not have ever been pregnant. In a small subset of patients, MMc was found in 45% of parous women, compared to 22% of nulliparous women. Thus, the same immunoregulatory mechanisms during pregnancy that allow increases in fetal microchimerism may also allow expansion of MMc.

The biological purpose of chimeric maternal cells is not known, but some clues can be derived from characterizing the phenotypes within tissues. We and others have discovered that maternal cells can engraft into a child's tissues [22,23]. We identified maternal cells in the thymus, heart, liver, kidney, lung, and pancreas using the FISH assay for X- and Y-chromosomes to identify female cells in tissues from males with inflammatory and non-inflammatory diseases [24]. MMc constituted 0.1 to 0.9% of parenchymal cells. To simultaneously identify and characterize the maternal cells, a technique was developed by which multiple phenotypic markers could be detected concurrently with FISH in the same cells of a tissue section. As circulating stem cells can have multilineage plasticity [25,26], we asked whether maternal cells can differentiate into tissue-specific phenotypes in her progeny. Female (maternal) cells within male tissues were characterized by simultaneous immunohistochemistry and FISH for X- and Y-chromosomes. Maternal cells expressed sarcomeric α-actin in the hearts of infants with neonatal lupus syndrome, indicating they had differentiated into cardiac myocytes or possibly fused with host cells [22]. Srivatsa, et al detected female cells (presumed maternal) in the tissues of four male newborns with congenital anomalies, but no inflammatory diseases, in the liver, thymus, thyroid and skin, but not in the spleen [23]. A controlled study to determine whether or not MMc is affected by inflammatory conditions in tissues has not been performed. How maternal cells function alongside host cells, and when allogeneic antigens on maternal cells may be recognized and attacked is not known.

### How does transplantation chimerism compare to MMc and FMc?

Chimerism, the state of cells from two genetically distinct individuals living within one body, can occur through multiple mechanisms. Stem cell transplantation, whether from bone marrow or peripheral stem cells, can lead to a spectrum of chronic inflammatory diseases called chronic GVHD [27-30]. Chronic GVHD has clinical similarities with some autoimmune diseases, including systemic sclerosis (SSc), primary biliary cirrhosis (PBC), Sjögren's syndrome, and some features of systemic lupus erythematosus (SLE) and myositis, although there are also pathological differences [27]. The chances that a patient will develop chronic GVHD are highly dependent upon the HLA genes of the donor and host. Thus, insights from transplantation chimerism contributed to the hypothesis that microchimerism and HLA-relationships of host and non-host cells are involved in spontaneously occurring autoimmune diseases. Clinical similarities of chronic GVHD and autoimmune disease are now considered in the context of cell transfer between fetus and mother during pregnancy. Comparison of fetal/maternal chimerism to transplantation chimerism must, however, take into account the significant differences in cell populations. In the case of fetal and maternal microchimerism, foreign cells are present at a frequency of less than 1%, in both hematopoietic and organ-specific lineages. In contrast, after stem cell transplantation, donor cells completely replace the hematopoietic system and may also constitute a small fraction of organ-specific cells.

### Is MMc is found in some autoimmune diseases?

MMc in the newborn is likely benign or may be beneficial, but may also transmit malignancy or cause GVHD [2,31-34]. Just as stem cell transplantation can lead to loss of tolerance to self antigens [27,30,35], natural transfer of maternal cells may lead to a child's loss of self-tolerance. MMc has been found increased in association with some autoimmune diseases. Long term MMc was first discovered in the peripheral blood of SSc patients and healthy subjects [17] and has since been identified in additional SSc patients [20] and in the target organs and blood in neonatal lupus syndrome (NLS) [22] and myositis [18,19]. In an early study, maternal DNA was found to be *increased *in prevalence and levels in patients with systemic sclerosis. MMc was initially detected in DNA isolated from peripheral blood mononuclear cells from 22% of healthy controls and 72% of women with SSc (OR 9.3, p = 0.001) [20]. The levels of MMc in the blood ranged from 0 to 68.6 gEq/million. The assay was then used to analyze MMc in organs from a woman who died of systemic sclerosis. MMc was found in tissues that were targets of disease in this patient, but also in tissues that were not involved. High levels of MMc were found in lung (757 gEq/million), heart (1489 gEq/million), spleen (466 gEq/million), and pancreas (704 gEq/million). Lower levels of MMc were also found in gut (39 gEq/million), and bone marrow (48 gEq/million). That the levels of MMc were 10 to 20-fold higher in the tissues than in the blood suggests that future studies into the mechanisms for the role of maternal cells in inflammatory disease may be best directed toward the parenchymal and immunological cells within the target organs.

We investigated parenchymal MMc in the context of an autoimmune disease that develops *in utero*, NLS [22]. Infants born to mothers with anti-SSA antibodies are at risk for developing NLS, with the life-threatening complication of inflammation of the atrial-ventricular node leading to congenital heart block [36]. Maternal (female) cells were detected and quantified in NLS and control male heart tissues by fluorescence *in situ *hybridization (FISH) for X- and Y-chromosome-specific sequences. In blinded studies, maternal cells were found in 15 of 15 sections of heart tissue examined from four NLS patients, ranging from 0.025% to 2.2% of host myocardial cells. Maternal cells were also found in two of eight control sections at lower levels (0.05–0.1%). Because recent studies in transplantation indicate that donor cells can differentiate into somatic tissue cells, we asked whether maternal cells transferred *in utero *have cellular plasticity. A small minority of maternal cells expressed the hematopoietic cell marker CD45. Eighty-six percent of maternal cells expressed sarcomeric α-actin, a specific marker for cardiac myocytes. These results suggest that differentiated tissue-specific maternal microchimerism can occur in the neonate. Thus, semi-allogeneic maternal cells could be the target of an immune response. Alternatively, maternal cells could contribute to a secondary process of tissue repair.

In older children, two groups have reported the presence of female cells (presumed maternal) in muscle biopsies from male patients with idiopathic myositis [18,19]. Age-matched controls who had biopsies for other muscle disorders carried significantly fewer female cells. Moreover, MMc in the blood was also increased in myositis patients, as detected by nested PCR assay for maternal HLA alleles not shared with the patient [19]. MMc has also been demonstrated in pityriasis lichenoides, where female cells in the form of keratinocytes were found in skin biopsies from males aged 2 to 13 years old [37]. No female hematopoietic or Langerhans cells were identified. The MMc in the skin was found in 11 of 12 patients at an average level of 99 per million host cells. Maternal cells were also found in some controls, but the levels were much lower (5 per million).

Two additional case reports have suggested that MMc may be common in chronic idiopathic inflammatory disease. In the first report maternal cells were found in an 11-year old boy with dermatitis, sclerodactyly, myositis and hepatitis with features of SLE and dermatomyositis [38]. By FISH for X- and Y-chromosomes, maternal nuclei were found in blood and in muscle. A second case report described a 21-year old man exposed to volatile chemicals in a tire factory who developed lymphocytic infiltrates and fibrosis in skin, lungs, and intestinal mucosa pathologically resembling chronic GVHD [39]. HLA typing revealed that he carried a SSc-associated gene DRB1*1104, but also had bi-directional compatibility at all HLA Class II loci with his mother. Maternal cells were found in his blood at a rate of 0.0017%. The patient also had active T lymphocyte activity to his mother. *In vitro *mixed lymphocyte cultures demonstrated a 2–3-fold increase in CD4+ and CD8+ T lymphocyte activation to maternal compared to unrelated donor antigen presenting cells, as demonstrated by increased HLA DR and CD25 expression. An environmental trigger has also been implicated in an animal study in which fetal microchimerism was found to be expanded and lead to sclerotic disease in response to polyvinyl chloride administration[40]. Thus, naturally-acquired maternal cells, normally present at a low level tolerogenic to the immune system, may be activated by an environmental stimulus to proliferate and expand in blood and affected tissues. At expanded numbers maternal cells would provide antigen levels adequate to overcome the threshold for activation of the host immune system. An alternative hypothesis suggests that maternal T lymphocytes may be reactive to the child's antigens.

T lymphocytes have been found in the affected skin of localized scleroderma patients, but also antigen presenting cells and B lymphocytes. [41] Thus, it is not clear what variety of allogeneic cell roles maternal cells may play in the blood or tissues of children with autoimmune diseases. MMc is not found in every suspected disease. Infantile hemangioma, hypothesized to be placental-derived maternal endothelial cells, was investigated for MMc. By FISH for X- and Y-chromosomes, no female cells were detected in hemangiomas from eight patients, although the amount of tissue assayed was not clear, and may have been too low to detect rare maternal cells [42].

Only one other functional study of MMc has been reported [43]. Chimeric maternal T lymphocytes were isolated from myositis patients and shown to react to the child's cells *in vitro *by producing IFN-γ. Maternal cells isolated from siblings did not react to the sibling's antigen presenting cells. Thus, although cells may change through culture conditions *in vitro*, maternal T lymphocytes may be recognizing the child's cells expressing non-shared MHC Class I or Class II molecules *in vivo*.

### How does the immune system tolerate MMc?

Why the host immune system does not eliminate allogeneic maternal cells is not known. The persistence of maternal cells in a child implies tolerance to maternal antigens, but studies thus far have demonstrated both tolerance and immunity. Tolerance to maternal antigens has been demonstrated in models of heart and skin allografts in the mouse. Maternal T lymphocytes in the lymph nodes, transferred either *in utero *or through nursing, have been correlated with maternal skin graft survival [44]. An independent study demonstrated a 40–90% reduction in splenocyte production of IL-2, IL-5, and INF-γ in response to antigen presenting cells expressing maternal MHC antigens in vitro [45].

In humans, T cell reactivity to maternal antigens has been reported to be decreased *in vivo*, allowing increased engraftment of maternal tissues when compared to semiallogeneic family or unrelated donors in some, but not all studies [46-48]*In vitro *studies have shown that although peripheral T lymphocyte reactivity to maternal antigens can be detected, it is reduced in some circumstances compared to reactivity to unrelated antigens [49-51] but not others [52,53] Moreover, the subset of cells responding to maternal antigens has been shown to be different from the cells responding to paternal antigens [54]. Whereas cells responding to paternal stimulator cells were enriched for CD3^+^/CD8^high ^cells, typical of allogeneic cytolytic T lymphocytes (CTL), responders to maternal stimulators were enriched in CD3^-^/CD8^dim ^cells, a phenotype typical of natural killer (NK) cells. Thus, there is evidence for CD8^+ ^lymphocyte tolerization to maternal cells, but the mechanisms involved are not known. B cell tolerance has been found in patients after multiple blood transfusions, but it is not known whether the B cells are directly tolerized by maternal antigens, or lack T cell help from tolerized T lymphocytes [55,56].

Alloreactive CTL and NK cells are crucial for the elimination of foreign cells after solid organ or stem cell transplantation [57]. NK cells, abundant in fetal blood, would be inhibited by HLA Class I molecules on maternal cells that are shared by the child, preventing elimination of MMc [58]. T lymphocytes, however, would be expected to react to maternal HLA molecules not inherited or shared by the child. The fetal immune system has been assumed to be too "immature" to reject maternal cells [59], but recent studies suggest that fetal CD8^+ ^T lymphocytes can develop specificity *in utero *[60]. Anti-maternal CTLs would therefore be expected to eliminate maternal cells.

Thus, mechanisms for developing tolerance to maternal antigens are not known, but thymic selection may be involved [61]. Donor dendritic cells engrafted into the thymus of the recipient can mediate renal allograft tolerance through clonal deletion of alloreactive thymocytes [62]. Moreover, intrathymic renal cells have delayed murine SLE nephritis [63]. Although the peripheral versus central mechanisms of tolerance remain to be explored, preliminary evidence suggests that maternal cells in the thymus may play a role in establishing central tolerance to maternal antigens [64].

### How do MHC alleles affect MMc and autoimmunity?

MMc is often found in healthy individuals. Therefore, if MMc has the potential to become pathogenic, additional environmental or genetic factors must be involved. The case of the tire factory worker suggests an environmental effect may activate immune responses to MMc [39]. The MHC may also play a role. MHC antigens direct an individual's ability to distinguish self antigens from foreign antigens. MHC molecules of donor and recipient determine transplantation tolerance. Each HLA class II molecule has two chains, α and β. HLA class II typing defines alleles (variant forms) of DQA1 and DPA1 (the genes that encode the α chains) and DRB1, DQB1, and DPB1 (the genes that encode the β chains). There is virtually no polymorphism (variability) of the DRα chain. Specific HLA alleles, especially DRB1 and DQA1, have been associated with autoimmune diseases [65]. Because microchimerism is associated with autoimmunity, the question arises: do particular MHC alleles affect the persistence or levels of MMc? One MHC class II allele, HLA DQA1*0501, has been associated with increased FMc and MMc in both the mother and the child [43,66]. How DQA1*0501 predisposes an individual to increased microchimerism is not known. In the mouse, fetal-maternal MHC compatibility has been suggested to mildly increase levels of MMc [67].

Because MHC Class II compatibility between donor and recipient is important in human GVHD as well as in a SLE-like GVHD in mice [68,69], we compared HLA compatibility between 30 male SLE patients and their mothers to 76 healthy males and their mothers [70]. Compared to controls, men with SLE had increased bi-directional compatibility (identical HLA alleles) with their mothers in HLA DRB1 allele families (OR 5.0, p = 0.006). The identity was also increased for specific DRB1 alleles (OR 4.0, p = 0.05). When analysis was limited to males who had SLE-associated HLA genes (encoding DR2 or DR3), there was an even greater increase in identity between SLE patients and their mothers in DRB1 families and DRB1 variant alleles (OR 7.2, p = 0.01 and OR 15, p = 0.018). The patients with SLE-associated HLA alleles also had increased compatibility with their mothers at DQA1 and DQB1. Whether this HLA matching allows MMc to persist at levels high enough to activate host T cells, or rather leads to cross-presentation of minor antigens remains to be discovered. It is not known whether maternal-fetal sharing of disease resistance alleles increases the protection from disease in healthy individuals who maintain normal levels of chimeric cells. Compatibility at minor histocompatibility antigens, also important for transplantation tolerance, has not been investigated in autoimmune diseases.

### What are additional sources of microchimerism derived during pregnancy?

In addition to cells from the mother, microchimerism could be derived from an older sibling, from a twin, or from a blood transfusion. Cells from an older sibling could persist in the mother for years after birth, and then be transferred to the fetus in a subsequent pregnancy. Because fetal cells transfer into the mother in the first weeks of gestation, spontaneous abortion (recognized or not) may lead to chimerism in a woman, which then could be transferred to the next fetus. Evidence for older sibling microchimerism is inconclusive so far. Twin-twin transfusion, however, has been established and occurs in up to 8% of twin pairs and 21% of triplet pairs [71]. Cells from a twin may completely replace the hematopoietic system [72]. The vanishing twin phenomenon, which may occur without recognition by mother or obstetrician, allows for the possibility of twin chimerism even in singlet pregnancies [73]. Blood transfusion can also lead to Mc. Transfusion after trauma-related hemorrhagic shock led to persistence of donor cells for at least 6–18 months [74,75]. In one study donor cells expressed CD4, CD8, CD15, and CD19, suggesting chimerism with a multipotent stem cell [74]. Transfusion-associated GVHD can also occur, with increased risk dependent on MHC compatibility of donor and host. [76] Thus, future studies of immune tolerance and autoimmune disease may consider the contributions of transfusions and maternal allogeneic antigens as well as paternal antigens that may be transmitted through older siblings or twins.

### Can animal models be used to study MMc?

Animal models are essential for investigations into the mechanisms of MMc regulation and treatments that may target MMc. There is evidence that in newborn mice MMc is a common phenomenon. In immunodeficient mice, MMc has been detected in hematopoietic organs (bone marrow, spleen, liver, lymph nodes, and thymus) and also non-lymphoid organs (heart, brain, and lung) [77-79]. Maternal cells were found as early as 12 days gestation, first in the thymus then later in other organs [77,79], and persisted as long as 24 weeks after birth [78]. In immunocompetent animals maternal cells have not been detected until later in gestation (day 16), and then mainly in the bone marrow and spleen [44,45,67,80-84]. Maternal cells may pass through the placenta into the fetus during pregnancy and may also be transferred through breast milk to the newborn pup [45,78,83]. Persistence of MMc after birth may depend on oral tolerance to maternal antigens transmitted through breast milk [45]. The levels of persistent MMc in brain and lymphoid tissues may be influenced by MHC compatibility of mother and pup [67]. Chimeric maternal cells have been shown to be functional, producing immunoglobulin, but do not regenerate the immune system for immunodeficient animals [78].

The mouse is a poor model for events occurring during human pregnancy, because of the physiological, immunological and anatomical differences between species [85,86]. For example, the placental hormone chorionic gonadotropin, which has an essential role in establishing and maintaining human pregnancy, is not produced by the mouse placenta. The mouse placenta is labyrinthine and hemotrichorial (three cell layers lay between maternal and fetal circulation: two layers of trophoblasts and one layer of syncytiotrophoblasts), whereas human placenta has villi and is hemochorial (containing only one layer of trophoblasts). Theoretically, the thinner barrier between maternal and fetal blood supplies in the mouse could lead to increased maternal-fetal cell transfer after injury or inflammation, but this is not known. In addition, most mouse models are inbred strains with homozygous and/or limited major histocompatibility (MHC) haplotypes, whereas in humans maternal-paternal MHC disparity is common, perhaps even required for successful pregnancy [87]. Many of the mouse chimerism studies were performed by blastocyst transfer rather than natural pregnancy, which may affect maternal-fetal cell trafficking [79-82]. As the mechanism of maternal-fetal cell transfer is not known, the influences of placental and genetic differences between humans and mice on the levels or pathogenicity of MMc cannot be determined. MMc has not been studied in other animals to my knowledge. FMc has been studied in pregnant non-human primates, where trends resemble those in humans, with increases during pregnancy and a rapid decrease after pregnancy [88]. How long FMc or MMc persists in primates, and how the primate immune system tolerates MMc is not known.

## Summary

MMc is commonly present in tissues and blood of patients with autoimmune disease. MMc is also found in healthy individuals, although at lower levels in some studies. The original hypothesis was that chimeric maternal or fetal T lymphocytes responding to host antigens led to chronic inflammation in a manner similar to GVHD, where maternal lymphocytes reacted to host antigens. The low frequency of maternal cells and the findings that maternal cells can differentiate into multiple hematopoietic and somatic cells suggests alternative hypotheses. Chronic inflammation may occur by host T lymphocyte activation in response to maternal cells within tissues. Injury or infection may upregulate maternal HLA expression, allowing the antigen load to exceed the T cell activation threshold for the otherwise tolerized host. The loss of tolerance to maternal antigens may extend to self antigens through epitope spreading, as after hematopoietic stem cell transplantation. Studies into the functional capabilities of maternal cells will be essential in understanding the biological significance of MMc in health and autoimmune disease.
